# When R‐Loops Go Awry: Genome Instability and Neurological Diseases

**DOI:** 10.1111/ejn.70513

**Published:** 2026-04-23

**Authors:** Nur Rasyiqin Rasli, Yu Katsuyama

**Affiliations:** ^1^ Division of Neuroanatomy, Department of Anatomy Shiga University of Medical Science Otsu Shiga Japan

**Keywords:** genome stability, neurodegenerative diseases, neurodevelopmental diseases, R‐loop, transcriptional stress

## Abstract

The basic structure of DNA is a double helix formed by base pairing between complementary strands. However, during transcription, RNA hybridizes with the template DNA, whereas the complementary DNA strand becomes displaced and remains unpaired. This process forms a DNA–RNA hybrid structure known as an R‐loop; similar structures can also occur in a non‐co‐transcriptional manner. In recent years, R‐loops have been reported to be involved in various cellular functions. However, when not properly regulated, they can compromise genomic DNA stability. R‐loops play roles in gene expression, DNA replication, and transcription termination. Dysregulation of R‐loop homeostasis has been implicated in various human diseases, including neurological diseases. In this review, we discuss the physiological and pathological roles of R‐loops, their related regulatory mechanisms controlling their formation and resolution, and their association with neurological diseases.

## Graphical abstract

DNA normally exists as a double helix formed by two complementary strands. During gene transcription, however, one strand of DNA can bind to RNA, causing the other DNA strand to be displaced. This creates a structure called an R‐loop. R‐loops play important roles in normal cellular processes such as gene expression, DNA replication, and transcription termination. However, if R‐loops are not properly controlled, they can lead to DNA instability and contribute to disease. This paper reviews the functions of R‐loops in the genome and the mechanisms that regulate them, with a particular focus on their involvement in neurological diseases.

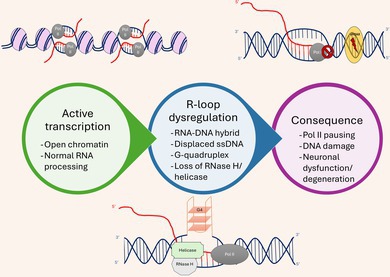

**Abbreviations**
ad
: Alzheimer's disease
ADAR1: Adenosine deaminase acting on RNA 1
AGS: Aicardi–Goutières syndrome
ALS: Amyotrophic lateral sclerosis
AOA2: Ataxia with oculomotor apraxia type 2
ARID1B: AT‐rich interaction domain 1B
ARK2N–CK2: Arkadia (RNF111) N‐terminal like PKA signalling regulator 2‐casein kinase II
ASD: Autism spectrum disorder
BAF: BRG1/BRM‐associated factor
BRCA: Breast cancer gene
C9orf72: Chromosome 9 open reading frame 72
CRISPR: Clustered regularly interspaced short palindromic repeats
CSB: Cockayne syndrome protein B
CSS: Coffin–Siris syndrome
CUT&RUN: Targeted DNA cleavage using nucleases
CUT&Tag: Targeted cleavage and tagging
DDR: Damage response
DDX: DEAD‐box helicases
DNA: Deoxyribonucleic acid
DRIP‐seq: DNA–RNA immunoprecipitation sequencing
DSBs: Double‐strand breaks
FA: Fanconi anemia
Fe–S: Iron–sulfur
FET: FUS, EWS/EWSR1, TAF15
FRDA: Friedreich's ataxia
FTD: Frontotemporal dementia
FXN: Frataxin
FXS: Fragile X syndrome
FXTAS: Fragile X‐associated tremor/ataxia syndrome
G4: Guanine quadruplex
GRN: Granulin precursor
HATs: Histone acetyltransferases
HD: Huntington's disease
HDACs: Histone deacetylases
HnRNP: Heterogeneous nuclear ribonucleoprotein
HRE: Hexanucleotide repeat expansion
HTT: Huntingtin
kDa: kiloDalton
lncRNAs: Long non‐coding RNAs
MAPT: Microtubule‐associated protein tau
mRNA: Messenger ribonucleic acid
mRNP: Messenger ribonucleoprotein
PCAF: p300/CBP‐associated factor
PD: Parkinson's disease
Pol II: RNA polymerase II
R‐ChIP: Reverse chromatin immunoprecipitation
RIAN‐seq: R‐loop identification assisted by nucleases and sequencing
RNA: Ribonucleic acid
RPA: Replication protein A
rRNA: Ribosomal RNA
SAMHD1: Sterile alpha motif and histidine‐aspartic acid domain containing protein 1
SCA: Spinocerebellar ataxia
SETX: Senataxin
ssDNA: Single‐stranded DNA
SWI/SNF: Switch/sucrose non‐fermentable
TARDBP: TAR DNA‐binding protein
TC‐NER: Transcription‐coupled nucleotide excision repair
TDP‐43: TAR DNA‐binding protein of 43 kDa
TERRA: Telomeric repeat‐containing RNA
Tip60: Tat‐interactive protein 60 kDa
TREX1: Three prime repair exonuclease 1
ZPR1: Zinc finger protein 1

## 1 Introduction

**FIGURE 1:**
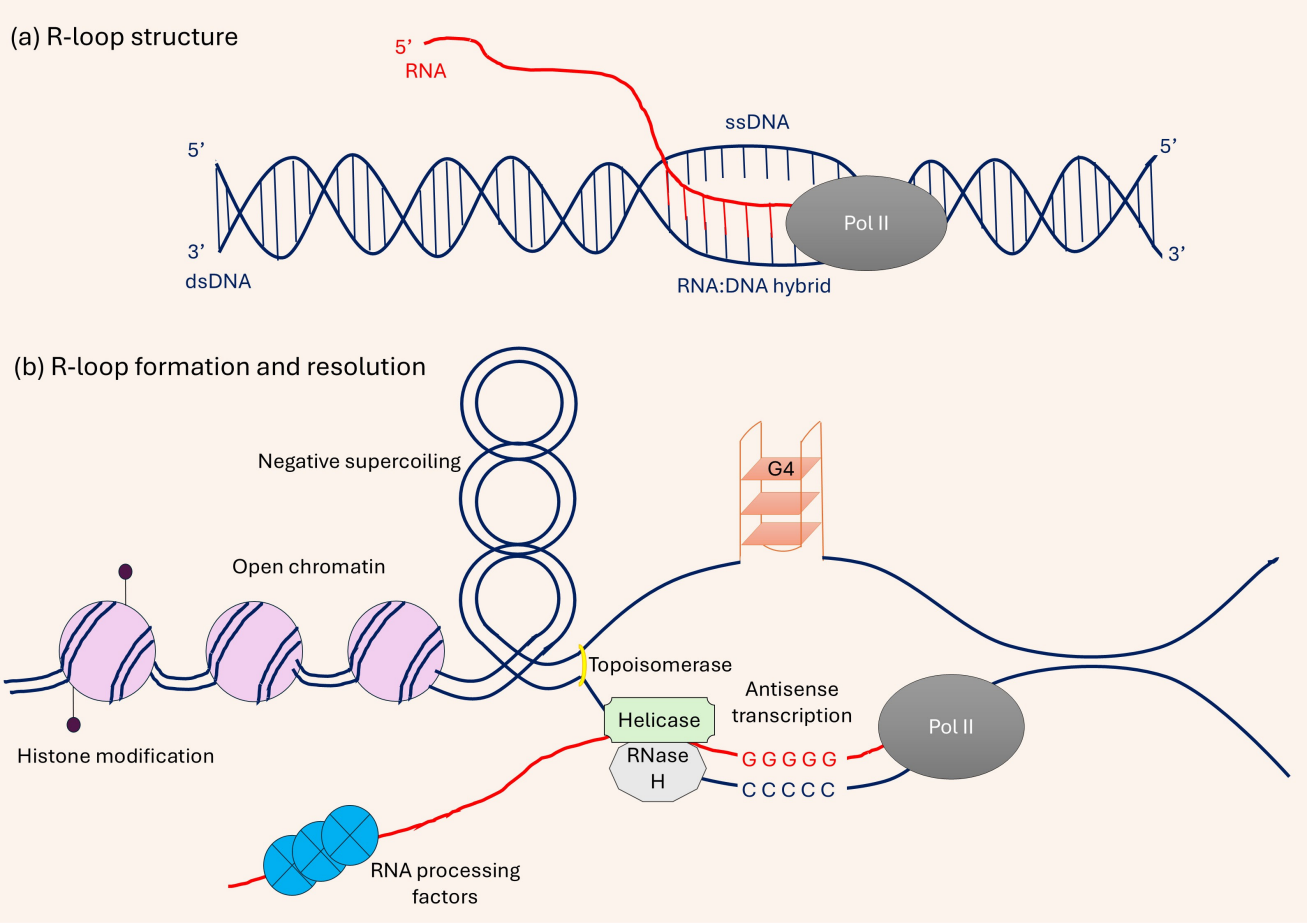
(a) Schematic representation of an R‐loop structure formed during transcription, consisting of an RNA–DNA hybrid and a displaced single‐stranded DNA region behind RNA polymerase II (Pol II). (b) Illustration of co‐transcriptional R‐loop formation and resolution. During active transcription in open chromatin, negative supercoiling behind RNA polymerase II promotes RNA–DNA hybrid formation, resulting in R‐loops characterized by a displaced single‐stranded DNA. The exposed strand can form G‐quadruplex structures, further stabilizing the R‐loop. Loss of RNase H activity and impaired helicase or RNA‐binding protein function prevents R‐loop resolution. Antisense transcription exacerbates hybrid formation, leading to transcriptional pausing or termination and genome instability.

The concept of R‐looping was first described in 1976 (Thomas et al. 1976), following the discovery of protein‐coding adenovirus genes which contained DNA sequences absent from the mature mRNA (Berk 2016; Kitchingman et al. 1977). R‐loops are three‐stranded nucleic acid structures formed co‐transcriptionally when the nascent RNA hybridizes with its complementary DNA template strand, generating a stable RNA–DNA hybrid and leaving the non‐template DNA strand displaced as single‐stranded DNA (ssDNA) (Figure 1a). Initially, R‐loops were thought to be non‐functional byproducts, without an active regulatory role in gene expression or genome maintenance. However, subsequent research revealed that these structures play important roles in transcription regulation and genome stability. Today, R‐loop formation is recognized as a physiologically important process, and abnormal regulation of R‐loops can lead to instability of the genome (Gan et al. 2011; Ginno et al. 2012; Wu et al. 2023).

Under physiological conditions, R‐loop structures form naturally during gene expression and are particularly stabilized in guanine‐ and cytosine‐rich or repetitive sequences due to stronger hydrogen bonding (Ginno et al. 2012). In addition, the high thermal stability of RNA–DNA hybrids can act as a physical barrier to replication fork progression, promoting fork stalling that may give rise to double‐strand breaks (DSBs) (Mudiyanselage et al. 2025; Sarni et al. 2022). Although R‐loops were initially thought to have negative effects on cells, accumulating evidence has shown that tightly controlled R‐loop formation plays important physiological functions. These functions include roles in transcription termination (Skourti‐Stathaki et al. 2011), DNA replication (Hamperl et al. 2017; Xu and Clayton 1996), immune diversification (Yu et al. 2003), and chromatin regulation (Ginno et al. 2012). One important role of R‐loops is their involvement in the process by which RNA polymerase terminates transcription independently of the DNA template (Loya and Reines 2016). In mitochondrial DNA, the accumulation of R‐loops at the origin of replication is important for initiating mitochondrial DNA replication (Xu and Clayton 1996).

R‐loops play important roles in immune function and chromatin regulation. During immunoglobulin class‐switch recombination, programmed R‐loop formation facilitates the generation of specific DNA structures, enabling targeted cytosine deamination and antibody diversification (Yu et al. 2003). Beyond immune processes, R‐loops contribute to chromatin maintenance (Chédin 2016) and are involved in regulatory regions associated with both repression and activation of gene expression (Herrera‐Moyano et al. 2014). These physiological roles indicate that controlled R‐loop formation is necessary for proper cellular functions.

In addition, R‐loops are involved in higher‐order nuclear organization, particularly through their contribution to biomolecular phase separation within organelles such as the nucleolus (Lawrimore et al. 2021; Zhou et al. 2020). In this context, RNA–DNA hybrids and associated RNA‐binding proteins help shape nucleolar architecture and regulate ribosomal biogenesis by modulating the surrounding chromatin environment (Abraham et al. 2020). R‐loops are also enriched at specific chromosomal regions, including centromeres, where they contribute to centromere function, chromatin organization, and chromosome segregation (Chakraborty et al. 2018; Chen et al. 2025). Similarly, R‐loop formation at telomeres regulates telomeric chromatin structure and maintenance, partly through interactions with telomeric repeat‐containing RNA (TERRA) and associated factors (Feretzaki et al. 2020). Additionally, R‐loops facilitate ribosomal RNA (rRNA) transcription initiation at ribosomal DNA loci and influence nucleolar function (Feng and Manley 2021). Long non‐coding RNAs (lncRNAs) can also form R‐loops at specific genomic loci to regulate chromatin states and gene expression (Arab et al. 2019). For example, the lncRNAs HOTAIR and APOLO associate with DNA through RNA–DNA hybrid formation, contributing to epigenetic regulation and transcriptional control (Ariel et al. 2020; Kalwa et al. 2016).

Proper regulation of R‐loop formation and resolution is crucial for maintaining genomic integrity and function. One of the most severe consequences of abnormal R‐loop accumulation observed in cellular and animal models is DSBs. R‐loop can disrupt genome stability through at least two different pathways. First, the displaced ssDNA strand within R‐loops becomes susceptible to enzymatic attack and chemical modification, including deamination by the APOBEC enzyme family (McCann et al. 2023). Second, the stability of RNA–DNA hybrids can disrupt replication fork progression, generating transcription‐replication conflicts (Kumar et al. 2021; Stoy et al. 2023). Persistent fork stalling may cause replication stress, fork collapse, and the formation of DSBs distal to the initial R‐loop structure (Mikolaskova et al. 2018; Rinaldi et al. 2021; Zhu et al. 2024).

Defective R‐loop resolution has been strongly implicated in cancer progression, where persistent transcription‐replication conflicts and impaired DNA repair contribute to mutagenesis, chromosomal rearrangements, and tumor development (Lam et al. 2020; Prendergast et al. 2020). Failures in R‐loop regulation can lead to immune deficiencies and autoimmune phenotypes, particularly through defects in class‐switch recombination, DNA repair pathways, and transcription‐associated genome maintenance (Sarkar et al. 2018; Yu et al. 2003). Additionally, inappropriate APOBEC activity during prolonged R‐loop exposure may cause deleterious mutations (Zong et al. 2023).

Neurons are non‐replicative cells in the human body and require proper and efficient mechanisms to maintain genomic integrity throughout a person's lifetime. However, due to their high energy consumption, high transcriptional activity, and limited capacity for regeneration, neurons are particularly vulnerable to genomic instability (Ihara et al. 2025). For example, DNA damage may arise through APOBEC3B‐mediated cytosine‐to‐uracil deamination (Argyris et al. 2007; McCann et al. 2023; Wang et al. 2009), or through replication‐associated DNA breaks caused by collision between R‐loops and replication forks, especially in a head‐on orientation (Hamperl et al. 2017). Although neurons do not normally undergo DNA replication, evidence indicates that neurons in several neurodegenerative and neurodevelopmental disorders can aberrantly re‐enter the cell cycle, initiating incomplete or abortive DNA replication programs that ultimately lead to DNA damage and cell death (Barrett et al. 2021; Ippati et al. 2021; Kruman et al. 2004; Marathe et al. 2015; Wu et al. 2024). Such pathological cell‐cycle re‐entry may create conditions permissive for replication‐associated R‐loop‐mediated genome instability in neurons.

R‐loop can also promote replication‐independent mechanisms that are particularly relevant in neurons, including transcription‐associated DNA breaks, impaired chromatin remodelling, and prolonged ssDNA exposure (Cristini et al. 2019; Sollier et al. 2014). These structures are increasingly recognized as endogenous sources of transcription‐associated stress in neurons, often occurring at neuron‐specific genes. Prolonged dysregulation of R‐loops can induce DNA damage, interfere with transcriptional processes essential for neuronal function, and disrupt chromatin structure, ultimately leading to neurological diseases. Although the specific mechanisms underlying each disease may vary, genomic instability, caused by DSBs and other related factors, has been implicated in neuronal dysfunction and increased susceptibility to neurological conditions (Dileep et al. 2023; Gospodinova et al. 2023; Perego et al. 2019; Sorrells et al. 2018; Westover et al. 2024).

This review focuses on the role of R‐loop in neuronal physiology and pathology, with emphasis on their contribution to genome instability and neurological diseases. First, we summarize the molecular mechanisms fundamental to R‐loop formation and resolution under physiological conditions. We then discuss how dysregulated R‐loop accumulation promotes DNA damage through both replication‐dependent and replication‐independent mechanisms. Finally, we describe the relationship connecting the defective R‐loop homeostasis to neurodevelopmental and neurodegenerative disorders.

## 2 Key Drivers of R‐Loop Formation

R‐loop formation is influenced by a wide range of molecular processes, including transcription by RNA polymerases (Broccoli et al. 2004; Lim et al. 2023; Zhang et al. 2017), DNA damage and repair pathways (Li, Smirnova, et al. 2024; Shih et al. 2022), RNA modification pathways (Gavaldá et al. 2013; Yang et al. 2019), chromatin organization (Konstantina et al. 2014), and transcriptional perturbations associated with genome engineering approaches such as CRISPR–Cas targeting (Laughery et al. 2018). In this review, we focus on selected, well‐characterized drivers of co‐transcriptional R‐loop formation—namely, RNA polymerase II (Pol II) dynamics, guanine‐rich DNA secondary structures, chromatin context, and RNA processing—because of their strong mechanistic relevance to transcription‐associated genome instability in neurons. Although negative supercoiling, histone modification, chromatin accessibility, and antisense transcription also contribute to R‐loop biology (Figure 1b), these mechanisms have been discussed extensively elsewhere and are therefore not systematically covered here (Crossley et al. 2019; García‐Muse and Aguilera 2019; Niehrs and Luke 2020; Santos‐Pereira et al. 2013).

In the classical model of gene expression, the nascent RNA transcript is efficiently displaced from the template DNA as transcription progresses (Boque‐Sastre et al. 2017). However, when transcription is transiently paused or stalled, particularly when the enzymatic activity of Pol II is compromised, co‐transcriptional R‐loop can be stabilized or persist (Rondón and Aguilera 2019). Importantly, even partial slowing of Pol II elongation, without complete arrest, can promote R‐loop accumulation by prolonging the residence time of nascent RNA near the DNA template, facilitating RNA–DNA hybrid formation (Han et al. 2025; Zatreanu et al. 2019). Such Pol II slowing frequently occurs at G:C‐rich sequences and repetitive DNA elements, which are intrinsically challenging to transcribe (Jonkers et al. 2014; Mimoso and Adelman 2023; Yuva‐Aydemir et al. 2019).

### 2.1 RNA Polymerase II (Pol II)

Pol II is a large multi‐subunit complex that synthesizes RNA from template DNA. Certain DNA lesions can act as physical barriers to Pol II, including oxidized bases, abasic sites, and bulky photoproducts such as cyclobutane pyrimidine dimers and 6–4 photoproducts, leading to transcriptional arrest or stalling (Kwei et al. 2004; Wang et al. 2018). In addition, G:C‐rich regions, trinucleotide repeats, and other repetitive sequences slow down Pol II elongation, increasing the probability of R‐loop formation even in the absence of overt DNA damage (Lin et al. 2010; Loomis et al. 2014; Skourti‐Stathaki et al. 2014). Such stalled Pol II complexes serve as signals for the activation of transcription‐coupled DNA repair pathways. The ARK2N–CK2 complex promotes recovery from transcriptional arrest by enhancing the interaction between Cockayne syndrome protein B (CSB) and lesion‐stalled Pol II, thereby facilitating the initiation of transcription‐coupled nucleotide excision repair (TC‐NER) (Luo et al. 2024). Mechanistically, the ARK2N‐CK complex stabilizes CSB at stalled transcription complexes, promotes Pol II remodelling, and allows access of downstream TC‐NER factors required for lesion recognition and repairs. Transcriptional interruptions commonly occur in promoter and terminator regions. Pol II frequently stalls at transcription initiation sites, which are known well‐established hotspots for co‐transcriptional R‐loop formation (Day et al. 2016). Although some RNA–DNA hybrids can form independently of ongoing transcription, for example those involving regulatory or structural RNAs such as lncRNAs (Ariel et al. 2020), the R‐loops discussed here are specifically co‐transcriptional in nature and arise during active Pol II transcription. Similarly, at transcription termination sites, delayed dissociation of Pol II from DNA creates hotspots at which R‐loops frequently form (Proudfoot 2016). Dysregulated persistence of co‐transcriptional R‐loops has been linked to transcriptional stress, impaired DNA repair, and genome instability observed in neurodegenerative disorders (Giannini et al. 2020; Walker et al. 2017; Wood et al. 2020). These pathological R‐loops can interfere with Pol II progression and DNA repair factor recruitment, thereby exacerbating the transcription–repair conflicts in neurons. Finally, it is important to note that many factors involved in Pol II‐associated R‐loop formation and resolution, including transcription‐coupled repair proteins and RNA helicases, also function in transcription, RNA processing, and genome maintenance, complicating interpretation of phenotypes observed upon their depletion.

### 2.2 Guanine Quadruplex (G4)

When guanine residues are abundant in the non‐template DNA strand during transcription, they can form a stable secondary structure called G4. In this structure, four guanine bases assemble into a planar configuration stabilized by Hoogsteen hydrogen bonding, with the assistance of monovalent cations such as potassium and sodium ions. Formation of G4s prevents reannealing with the template strand, thereby stabilizing the R‐loop. Accordingly, G4s frequently co‐localize with R‐loops and are enriched near gene promoters (Lipps and Rhodes 2009). Rather than acting as strong intrinsic DSB hotspots in unstressed cells, R‐loops and associated G4 structures are thought to create transcription‐associated regions of genomic vulnerability. These regions are susceptible to DNA damage under conditions of impaired R‐loop resolution or defective DNA repair pathways (Wulfridge and Sarma 2024).

### 2.3 Epigenomic Context

Chromosomes are composed of DNA and histone proteins, and their structure dynamically changes to regulate gene expression. R‐loops arise predominantly as a co‐transcriptional intermediate during Pol II activity and are therefore inherently enriched in euchromatic regions, where chromatin is open and transcriptionally active. In such regions, where DNA is more accessible, transcriptional regulatory proteins can more easily interact with DNA, increasing the likelihood of R‐loop formation (Belotserkovskii et al. 2018; Li et al. 2004). In contrast, R‐loop formation is less common in heterochromatin, where the chromatin is more condensed and transcription is repressed.

Besides that, chromatin is shaped by epigenetic modifications that refine transcriptional dynamics and genome stability. Histone acetylation, mediated by histone acetyltransferases (HATs), plays an important role in modulating chromatin accessibility and Pol II elongation dynamics. It also facilitates the recruitment of DNA repair proteins to sites of DNA damage (Sharma and Hendzel 2019). Acetylation neutralizes positively charged lysine residues on histones, loosening the interaction between histones and DNA and reducing nucleosome‐induced physical barriers to Pol II progression. This chromatin relaxation increases transcriptional elongation rate, limiting Pol II pausing and backtracking and eventually suppresses co‐transcriptional R‐loop formation.

Acetylation of histones H3 and H4 by specific HATs, such as PCAF (p300/CBP‐associated factor) and Tip60, relaxes chromatin structure, thereby preventing transcriptional arrest and maintaining genomic stability (Lee et al. 2024; Li, Yang, et al. 2024). Importantly, HATs primarily function to prevent R‐loop accumulation rather than acting as mediators of R‐loop resolution after formation (Cañas et al. 2022; Lee et al. 2024). Through this mechanism, HAT activity indirectly maintains the genome stability by limit the R‐loop‐induced transcription–replication conflicts and DNA damage. Conversely, deacetylation of histones by histone deacetylases (HDACs) tightens chromatin structure, slows Pol II elongation, enhances polymerase pausing, leading to transcriptional stress and increased R‐loop accumulation (Nie et al. 2024).

The chromatin environment is not solely defined by its structural state but is also shaped by epigenetic modifications that fine‐tune transcriptional activity and DNA accessibility. Beyond transcriptional activity, epigenetic features of euchromatin play a critical role in modulating R‐loop stability and resolution. Epigenetic modifications, including DNA methylation and various histone marks, influence DNA accessibility to the transcriptional machinery and thereby affect R‐loop formation (Ginno et al. 2013). For example, the H3K36me3 histone modification promotes transcription while also facilitating helicase activity, helping prevent prolonged R‐loop persistence (Uruci et al. 2021). Disturbance of this chromatin‐mediated regulation, caused by altered histone modifications or impaired recruitment of R‐loop–resolving machinery, may shift R‐loops from temporary transcriptional intermediates to stable genomic lesions (Bayona‐Feliu et al. 2017; García‐Pichardo et al. 2017; Lee et al. 2024). Such persistent R‐loops can contribute to transcription‐associated DNA damage and genome instability.

### 2.4 RNA Processing and Nuclear Export

Efficient RNA processing and nuclear export represent important mechanisms that control abnormal R‐loop accumulation. Newly synthesized RNA must be rapidly packed into ribonucleoprotein (RNP) complexes to prevent re‐hybridization with the DNA template strand (Santos‐Pereira et al. 2013). Failure of this process promotes the persistence of RNA–DNA hybrids and the exposure of ssDNA, thereby increasing genome instability (Gómez‐González et al. 2011). The THO/TREX complex plays a central role in coupling transcription with mRNA processing and nuclear export, ensuring proper assembly of messenger RNP (mRNP) complexes (Huertas and Aguilera 2003). Disruption of THO/TREX components leads to defective RNA packaging, impaired transcription elongation, and increased R‐loop formation, which in turn promotes transcription‐associated DNA damage (Dominguez‐Sanchez et al. 2011). These observations indicate that RNA processing and export machineries serve as critical safeguards against R‐loop‐mediated genomic instability, functioning alongside DNA repair pathways to preserve transcriptional fidelity and cellular homeostasis. Because RNA processing and export factors act at multiple stages of gene expression, defects attributed to R‐loop accumulation may reflect combined effects on transcription, RNA maturation, and nuclear trafficking rather than dysregulation alone.

## 3 Key Players in R‐Loop Resolution

Physiological R‐loops are transiently formed during key biological processes such as transcription termination, immunoglobulin class switch recombination, mitochondrial DNA replication, and regulation of gene expression at CpG‐rich promoters (Ginno et al. 2012; Lombraña et al. 2015; Skourti‐Stathaki et al. 2011; Yu et al. 2003). In these contexts, R‐loops perform normal genome functions by regulating chromatin structure and assisting an accurate transcriptional process. Although R‐loops are formed to serve specific functions, they must be properly resolved afterward to prevent genome instability (Amon and Koshland 2016; Saha et al. 2022; Ihara et al. 2025). Thus, the cellular impact of R‐loops is highly context‐dependent, with different regulatory mechanisms working together to maintain a balance between their beneficial and harmful effects. Multiple proteins and pathways work together to resolve R‐loops and preserve genomic stability (Figure 1).

### 3.1 RNase H

RNA bound to the template DNA is selectively degraded by RNase H1 and RNase H2 to resolve R‐loops (Allison and Wang 2019; Zhao et al. 2018). RNase H1 and RNase H2 are non‐redundant enzymes with complementary but distinct cellular functions. RNase H1 primarily degrades the RNA strand within the R‐loop, allowing the complementary DNA strands to re‐anneal (Li et al. 2021). RNase H2, in addition, removes ribonucleotides that are mistakenly incorporated into DNA during replication (Chon et al. 2013). Loss of RNase H2 activity, rather than RNase H1, is strongly associated with pathological R‐loop accumulation and defective DNA repair, leading to transcription‐replication conflicts and DNA damage (Amon and Koshland 2016). Conditional loss of RNase H2 in mouse brain results in neuronal and astrocyte defects, along with evidence consistent with R‐loop accumulation (Bartsch et al. 2018; Downing et al. 2021).

### 3.2 Topoisomerases

The torsional stress generated during transcription and replication is typically resolved by topoisomerases, which prevent the accumulation of excessive supercoiling (Bermejo et al. 2012; Manzo et al. 2018). During transcription, positive supercoiling accumulates ahead of the progressing Pol II complex, whereas the DNA behind it becomes underwound. If not properly resolved, these topological constraints can cause RNA to re‐anneal the DNA template strand, promoting R‐loop formation and transcriptional stalling (Patel et al. 2022). Topoisomerase I relieves transcription‐induced supercoiling by introducing transient ssDNA breaks, whereas topoisomerase II resolves higher‐order topological stress by generating transient DSBs. These controlled cleavage–religation reactions are essential for relieving supercoiling, thereby reducing the likelihood of R‐loop formation, preventing continuous DNA damage, and maintaining genomic integrity (Cho and Jinks‐Robertson 2018; Yadav et al. 2016). Failure of topoisomerase activity can convert these transient breaks into pathological DNA lesions and exacerbate R‐loop accumulation. In neurons, pharmacological inhibition of topoisomerase I by topotecan leads to unresolved transcription‐associated supercoiling, resulting in increased R‐loop formation (Powell et al. 2013).

### 3.3 Helicases

Helicases help eliminate R‐loops by unwinding the hydrogen bonds between nucleic acid strands. SETX helicase specifically resolves R‐loops formed during transcription termination and at sites of transcription‐replication conflicts (Hasanova et al. 2023). Importantly, both loss‐of‐function and activating mutations in SETX differentially impact R‐loop dynamics: SETX deficiency leads to pathological R‐loop accumulation, DNA damage, and neurodegenerative phenotypes, whereas activating mutations are associated with altered transcriptional stress responses and aberrant R‐loop resolution (Cohen et al. 2018; Hasanova et al. 2023; Jurga et al. 2021). Members of the DExD/H‐box family of DDX helicases, DHX9 (Cristini et al. 2018), DDX23 (Sridhara et al. 2017), DDX19 (Hodroj et al. 2017), DDX1 (Amorim et al. 2024), and DDX21 (Song et al. 2017), also contribute to R‐loop resolution, either by disrupting RNA–DNA hybrids or by preventing their formation (Mersaoui et al. 2019; Song et al. 2017). Aquarius helicase helps maintain genome stability by preventing R‐loop–associated DNA damage and promoting homologous recombination repair of DNA DSBs through both DSB processing factor‐dependent and independent mechanisms (Sakasai et al. 2017). Additional helicases implicated in R‐loop suppression include BLM/yeast Sgs1 (Chang et al. 2017), FANCM (Pan et al. 2019), and PIF1 (Tran et al. 2017), highlighting a helicases network that protect the genome stability.

### 3.4 DNA Repair and Recombination Factors

The tumor suppressors BRCA1 and BRCA2 play central roles in homologous recombination‐mediated repair at R‐loop‐associated DNA lesions. BRCA1 has been shown to localize to genomic regions containing R‐loops, where it promotes their resolution and prevents transcription‐associated DNA damage, partly by recruiting RNA‐processing and DNA repair factors (Chiang et al. 2019; Hatchi et al. 2015; Vohhodina et al. 2021). Loss of BRCA1 results in R‐loop accumulation, replication stress, and increased DSBs (Patel et al. 2023; Vohhodina et al. 2021). Similarly, BRCA2 suppresses R‐loop accumulation by stabilizing stalled replication forks and facilitating RAD51 loading, thereby reducing transcription‐replication conflicts and preserving genome integrity (Bhatia et al. 2014). Replication protein A (RPA), as an ssDNA‐binding complex, protects exposed ssDNA generated during transcription or replication stress, preventing secondary structure formation and aberrant RNA–DNA hybrid stabilization (Feng and Manley 2021; Nguyen et al. 2017). The Fanconi anemia (FA) proteins, including FANCD2 and FANCA, are recruited to sites of transcription‐replication conflicts, where they facilitate processing of stalled replication forks and DNA interstrand crosslinks (Schwab et al. 2015). FA pathway deficiency leads to R‐loop accumulation, replication stress, and genome instability, providing a direct link between defective R‐loop resolution and the clinical manifestations of FA (García‐Rubio et al. 2015; Okamoto et al. 2019; Olazabal‐Herrero et al. 2024). Although BRCA‐ and FA‐pathway proteins have been primarily studied in proliferating cells, their roles in maintaining transcription‐associated genome stability may also be relevant in post‐mitotic neurons, where unresolved R‐loops have been implicated in genome instability and neurodegeneration (Bhatia et al. 2014; Racca et al. 2021; Alonso and Noordermeer 2021).

## 4 R‐Loops as Drivers of Genome Instability in the Nervous System

Neurons are post‐mitotic and therefore rely heavily on efficient DNA repair and high transcriptional fidelity to preserve genome integrity. R‐loops have emerged as important regulators of neuronal genome function. Within an R‐loop, the displaced non‐template DNA strand is particularly vulnerable to oxidative damage, enzymatic modification, and nucleolytic cleavage. This vulnerability is exemplified by activation‐induced cytidine deaminase‐mediated deamination of exposed ssDNA in yeast R‐loop (Cañas et al. 2022), subsequent processing by the base excision repair pathway leading to DNA fragility (Su and Freudenreich 2017), and cleavage by structure‐specific nucleases that generate strand breaks (Cristini et al. 2019). Neurons are especially susceptible to such damage owing to their high metabolic activity and elevated levels of reactive oxygen species (Foret et al. 2024; Ma et al. 2023). Persistent ssDNA exposure therefore promotes both single‐ and DSBs, contributing to the accumulation of DNA damage commonly observed in neurodegenerative disorders (Cristini et al. 2019; Thongthip et al. 2022). In addition, impaired recruitment of activity of DNA repair factors at R‐loop‐prone genome further exacerbates genome instability in neuronal cells (Sorrells et al. 2018).

RNA‐binding proteins that associate with nascent transcripts play critical roles in preventing aberrant R‐loop persistence in neurons. Among these, members of the FET protein family, including FUS and EWS, function as co‐transcriptional regulators that limit RNA re‐hybridization to the DNA template and promote efficient RNA processing (Luo et al. 2015; Thompson et al. 2023). Through interactions with Pol II, chromatin, and RNA‐processing machineries, FET proteins suppress excessive R‐loop accumulation and facilitate timely recruitment of DNA repair factors to sites of transcription‐associated DNA damage (Hill et al. 2016; Singatulina et al. 2019). Loss of nuclear FET protein function, as observed in several neurodegenerative disease contexts, results in increased R‐loop persistence, prolonged ssDNA exposure, and activation of DNA damage response (DDR) pathways, thereby enhancing transcription‐associated genome instability in post‐mitotic neurons (Naumann et al. 2018; Wang et al. 2013).

Beyond their impact on genome stability, R‐loops also play important regulatory roles in neuronal gene expression. Neurons exhibit exceptionally high transcriptional activity and depend on precise temporal and spatial regulation of gene expression to support synaptic function, plasticity, and activity‐dependent responses (Flavell and Greenberg 2008). R‐loops formed at promoters, enhancers, and gene bodies can modulate Pol II pausing, elongation, and chromatin accessibility, thereby shaping transcriptional outcomes (Chen et al. 2017; Tan‐Wong et al. 2019; Zhang et al. 2024). In certain contexts, R‐loops have been shown to facilitate transcriptional activation of neuronal genes (Akiki et al. 2024). Disruption of these regulatory functions may therefore contribute to aberrant expression of genes involved in synaptic signalling, neuronal connectivity, and cellular stress responses.

Finally, R‐loops participate in transcriptional termination in neurons, a process that is essential for preventing transcriptional interference and maintaining proper genome organization. Formation of R‐loops downstream of polyadenylation sites promotes Pol II pausing and release, thereby ensuring efficient transcriptional termination (Skourti‐Stathaki et al. 2014). Failure to properly resolve these structures can result in transcriptional readthrough, collision with adjacent transcriptional units, and the accumulation of DNA damage.

## 5 R‐Loop in Neurodegenerative and Neurodevelopmental Disorders

In general, dysregulation of R loops has been associated with neurodegenerative and neurodevelopmental diseases through transcriptional stress, DNA damage, and genomic instability. However, their contribution to disease phenotypes in neurons is still being actively investigated. Importantly, R‐loops are now recognized as “Janus‐faced” structures that can exert both detrimental and context‐dependent protective roles, including facilitation of DSB repair (Marnef and Legube 2021; Ngo et al. 2021).

In neurons, the balance between physiological and pathological R‐loop formation appears particularly fragile, due to high transcriptional activity, long gene architecture, and limited DNA repair capacity. A “long gene” is commonly referred to as a gene in the genome that is significantly larger than average (over 100 kilobases), and is often associated with complex functions, particularly in the brain and during development (King et al. 2013; Zylka et al. 2015). Because of their size and sustained transcriptional activity, long genes are especially susceptible to transcription‐associated stress, including R‐loop formation and DNA damage (Helmrich et al. 2011).

**FIGURE 2:**
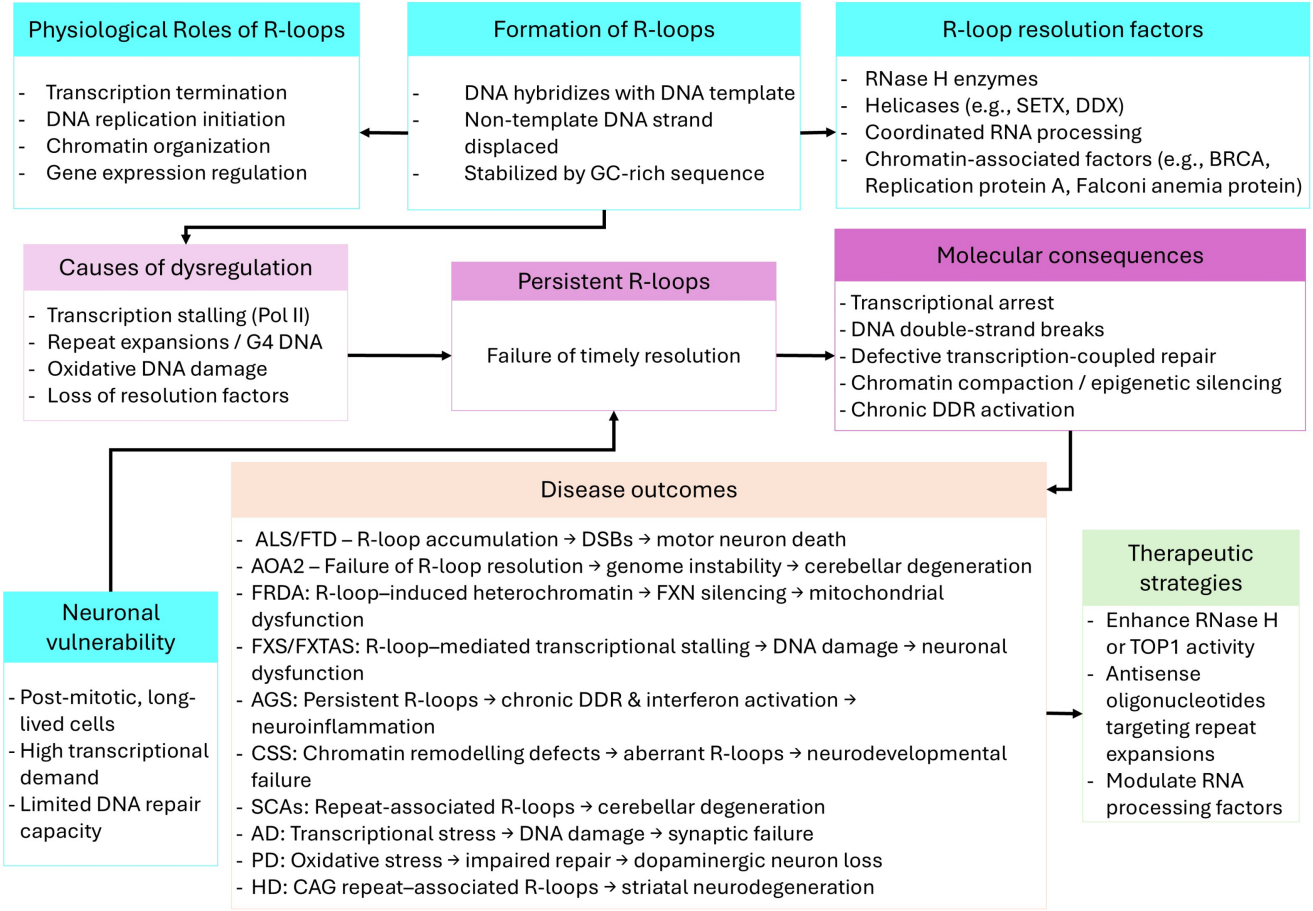
Schematic representation of R‐loop dysregulation in neuronal cells. R‐loops are physiological transcription‐associated structures that contribute to gene regulation and genome organization when properly controlled. Their formation is regulated by RNA‐processing and resolution factors, including RNase H enzymes, senataxin (SETX), and topoisomerases. In neurons, high transcriptional activity combined with limited DNA repair capacity increases vulnerability to R‐loop dysregulation. Persistent or unresolved R‐loops can cause transcriptional stalling, DNA double‐strand breaks, chromatin alterations, and impaired transcription‐coupled repair, ultimately leading to neuronal dysfunction and neurodegeneration. Disease‐associated examples illustrate how R‐loop–mediated genome instability contributes to the pathology of both neurodegenerative and neurodevelopmental disorders.

Neurons are post‐mitotic and non‐dividing cells and rely heavily on long‐term genome maintenance mechanisms, predominantly using the transcription‐coupled and non‐homologous end‐joining pathways while lacking replication‐associated repair mechanisms (Keqin and Sandra Peña De 2002; Welty et al. 2018; Wu et al. 2021). Persistent R‐loop accumulation at long, highly transcribed neuronal genes has been proposed to worsen genome stability and increase mutation over time (Provasek et al. 2022; Sun et al. 2023). Although genomic instability is a well‐established feature of neurodegenerative diseases such as Huntington's disease (HD) and ALS, the extent to which R‐loop dysregulation directly drives this instability remains unclear (Maiuri et al. 2021; Ziff et al. 2023). Currently, no curative therapies exist for the neurological diseases discussed here, and patients are typically left to manage lifelong symptoms and complications. Dysregulation of R‐loops has been implicated in the pathogenesis of these diseases (Figure 2).

### 5.1 Ataxia With Oculomotor Apraxia Type 2 (AOA2)

AOA2 is a neurodegenerative disease caused by mutations in the *SETX* gene, which have been strongly associated with altered R‐loop homeostasis and genome instability. This disease is characterized by peripheral neuropathy, cerebellar atrophy, and progressive cerebellar ataxia sometimes accompanied by oculomotor ataxia (Anheim et al. 2009) and is inherited in an autosomal recessive manner (Le Ber et al. 2004). In normal cells, SETX collaborates with other factors such as XRN2, RNase H, and topoisomerases to ensure effective resolution of R‐loops during gene expression. In addition to its role in R‐loop resolution, SETX is also involved in transcription‐coupled DNA repair and RNA processing events, including splicing and 3′‐end formation of transcripts, highlighting its multifunctional nature (Elodie et al. 2015; Hasanova et al. 2023).

In AOA2, however, mutations on the *SETX* gene are distributed throughout the coding region, with the majority being frameshift or nonsense mutations. These mutations are likely to severely impair SETX protein function, including its helicase activity (Nakamura et al. 2009). Loss of SETX function has been shown to result in increased accumulation of unresolved R‐loops, which are thought to contribute to genomic instability rather than representing the sole pathogenic mechanism (Jurga et al. 2021; Becherel et al. 2013; Wu et al. 2025). Although neurons are post‐mitotic and do not undergo DNA replication, persistent R‐loop accumulation in these cells can trigger cellular stress responses, interfere with transcription, and compromise genomic stability.

In an experimental model, disruption of the *SETX* gene has been shown to cause persistent DSBs, defects in Rad51 filament disassembly, and increased accumulation of R‐loops (Becherel et al. 2013). In addition, *SETX* depletion has been linked to impaired autophagy progression, resulting in the accumulation of ubiquitinated proteins and reduced capacity to clear protein aggregates. These findings suggest that multiple SETX‐dependent pathways may converge to enhance DNA damage signalling, promote further R‐loop accumulation and genomic stress (Richard et al. 2021). Collectively, these observations support a model in which dysregulated R‐loop metabolism represents a major contributing factor, but not the exclusive cause, of AOA2 pathogenesis (Richard et al. 2013; Roda et al. 2014; Yüce and West 2013).

### 5.2 Amyotrophic Lateral Sclerosis (ALS)–Frontotemporal Dementia (FTD) Spectrum

ALS and FTD are now widely recognized as forming a disease continuum rather than representing entirely distinct neurological entities. This concept is supported by overlapping clinical features, shared genetic etiologies, and convergent molecular pathologies. Mutations in C9orf72 constitute the most common genetic cause of both familial ALS and FTD, whereas pathological aggregation and nuclear depletion of TDP‐43 are observed in most ALS cases and in a substantial proportion of FTD patients. Together, these shared features suggest that common mechanisms of genomic instability and RNA dysregulation contribute to neurodegeneration across the ALS–FTD spectrum.

ALS is typically characterized by the progressive loss of neurons in the brain that project to the spinal cord, as well as motor neurons that control voluntary movements such as chewing, walking, and speaking (Nijssen et al. 2017). Although the exact etiology of ALS is not fully understood, genetic mutations and molecular processes that cause neuronal dysfunction have been reported to contribute to disease pathogenesis. An important hallmark shared between ALS and FTD is pathology involving TAR DNA‐binding protein of 43 kDa (TDP‐43).

Under physiological conditions, TDP‐43 is predominantly localized in the nucleus, where it plays a critical role in maintaining R‐loop homeostasis. TDP‐43 suppresses excessive R‐loop formation by promoting proper mRNP assembly on nascent transcripts, thereby limiting RNA re‐hybridization to the DNA template (Xiao et al. 2011). In addition, TDP‐43 facilitates the resolution of pre‐formed R‐loops by recruiting RNA‐processing and R‐loop–resolving factors, including RNase H1 and RNA helicases (Hill et al. 2016; Wood et al. 2020).

However, under disease conditions, TDP‐43 is mislocalized from the nucleus to the cytoplasm. This mislocalization, observed in both ALS and FTD, is often accompanied by protein aggregation, leading to a loss of its nuclear functions. Depletion of nuclear TDP‐43 reduces its ability to regulate transcription and resolve R‐loops (Hou et al. 2024). Consequently, aberrant R‐loops accumulate at transcriptionally active genes, leading to the formation of DNA DSBs. These DNA lesions activate the DDR, which, if not properly resolved, can trigger cell death pathways in vulnerable neuronal populations.

Experimental studies have demonstrated that depletion or cytoplasmic mislocalization of TDP‐43 increases R‐loop levels, particularly at promoters and transcription start sites, and is accompanied by elevated γH2AX signals, a marker of DNA damage. Importantly, overexpression of RNase H1 in these models rescues the DNA damage phenotype, confirming that the observed defects are specifically attributable to R‐loop accumulation (Giannini et al. 2020).

Additional genetic evidence further supports a role for defective R‐loop resolution in ALS. Familial ALS type 4 (ALS4) is an autosomal dominant form of ALS that can be differentiated from other subtypes by the absence of respiratory or bulbar paralysis. ALS4 therefore provides compelling evidence linking impaired R‐loop resolution to motor neuron degeneration. ALS4 has been shown to be associated with heterozygous mutations in the *SETX* gene, as well as with dysfunction of zinc finger protein 1 (ZPR1), which recruits SETX to R‐loop–containing genomic regions (Kannan et al. 2022). Together, SETX and ZPR1 form a functional R‐loop–resolution axis, and disruption of either component promotes R‐loop persistence and genomic instability in ALS4.

FTD shares overlapping molecular features with ALS, particularly in cases linked to C9orf72 repeat expansions. FTD is a highly heritable neurodegenerative disease characterized by progressive degeneration of the frontal and temporal lobes of the cerebral cortex, with symptom onset typically occurring between the ages of 45 and 64 (Moore et al. 2020). Although autosomal dominant mutations in C9orf72, GRN, and MAPT have been identified in familial FTD, R‐loop–associated genomic instability is most strongly linked to C9orf72 repeat expansions, which represent the most common genetic cause of both familial and sporadic FTD (Schönecker et al. 2022; Sha et al. 2012). Current evidence suggests that expanded GGGGCC repeats at the C9orf72 locus promote R‐loop formation due to their high GC content and transcriptional instability. These GC‐rich repeats facilitate stable R‐loop formation during transcription, in which repeat‐containing RNA hybridizes with the template DNA strand, displacing the non‐template strand and generating persistent RNA–DNA hybrids (Gossye et al. 2020; Reddy et al. 2014). Prolonged R‐loop formation at the C9orf72 locus negatively affects genomic stability by interfering with transcriptional elongation, ultimately leading to transcriptional arrest (Kang et al. 2025; Konopka and Atkin 2018; Reddy et al. 2014).

Although R‐loops are known to destabilize repetitive genomic loci, direct evidence that they actively drive further repeat expansion in neurons remains limited. This suggests that R‐loops primarily arise because of repeat expansion and function as amplifiers of genomic instability rather than as initiating events (Haeusler et al. 2014). In addition to inducing DNA damage, R‐loops formed at expanded C9orf72 repeats can sequester RNA‐binding proteins required for mRNA splicing and transport, disrupt autophagic processes, and impair RNA metabolism (Jiang and Ravits 2019; Kumar et al. 2017; Porta et al. 2015). Collectively, these mechanisms link R‐loop accumulation at expanded repeat loci to genomic instability and neurodegeneration across the ALS–FTD spectrum.

### 5.3 Friedreich's Ataxia (FRDA)

FRDA is a rare inherited neurodegenerative disease characterized by spinocerebellar ataxia (SCA), pyramidal muscle weakness, loss of deep sensation, hypertrophic cardiomyopathy, skeletal abnormalities, impaired reflexes, loss of proprioception, and dysarthria. This disease is caused by a GAA trinucleotide repeat expansion in the first intron of the *FXN* gene, located on chromosome 9q21.11, which encodes the mitochondrial protein frataxin (Reetz et al. 2021). Although frataxin deficiency primarily disrupts mitochondrial iron–sulfur (Fe–S) cluster biogenesis and cellular redox balance (Lu and Cortopassi 2007), increasing evidence suggests that transcription‐associated genomic instability also contributes to disease pathogenesis (Groh et al. 2014; Li et al. 2016; Punga and Bühler 2010).

Under normal physiological conditions, frataxin regulates iron homeostasis by limiting mitochondrial iron accumulation and supporting the synthesis of Fe–S clusters. In FRDA, however, expanded GAA repeats within the *FXN* gene promote the formation of stable transcription‐associated R‐loops, consisting of RNA–DNA hybrids and displaced ssDNA. These stable R‐loops hinder Pol II elongation, leading to transcriptional stalling and reduced FXN RNA expression (Groh et al. 2014). Prolonged R‐loop formation at the expanded GAA repeat region further contributes to chromatin remodelling and transcriptional repression. Specifically, R‐loop formation has been associated with heterochromatinization of the *FXN* locus, including increased histone deacetylation, DNA methylation, and histone trimethylation such as H3K9me3 and H3K27me3 (Crossley et al. 2019; Groh et al. 2014; Sherzai et al. 2020; Trabzuni 2009). Collectively, these findings identify R‐loop as a key molecular intermediate linking repeat expansion with epigenetic silencing and transcriptional dysfunction in FRDA (De Biase et al. 2009; Rodden et al. 2021).

### 5.4 Spinocerebellar Ataxia (SCA)

SCAs represent a heterogeneous group of inherited neurodegenerative disorders characterized by progressive cerebellar degeneration, motor incoordination, and neuronal loss (Ashizawa et al. 2013; Jacobi et al. 2013; Schelhaas and Van De Warrenburg 2005). Many SCAs are caused by repeat expansion mutations, including CAG and other trinucleotide or pentanucleotide repeats, which are inherently prone to transcriptional instability (Holmberg et al. 1998; Lindblad et al. 1996; Silveira et al. 2002). Expanded repeat tracts promote R‐loop formation due to their repetitive nature, high GC content, and inefficient transcriptional elongation. R‐loops formed at these loci can obstruct Pol II progression, induce chromatin remodelling, and generate DNA damage.

Moreover, evidence from multiple experimental models suggests that transcription‐associated R‐loop contributes to repeat instability, epigenetic silencing, and neuronal dysfunction (Kaalak et al. 2014; Su and Freudenreich 2017; Wongsurawat et al. 2011). Impaired resolution of R‐loops may interfere with transcription‐coupled DNA repair pathways and exacerbate DNA damage accumulation in cerebellar neurons, which are particularly sensitive to genomic stress. These findings indicate that R‐loop dysregulation represents a unifying molecular mechanism across distinct SCA subtypes, linking repeat expansion, transcriptional stress, and progressive neurodegeneration.

### 5.5 Fragile X Syndrome (FXS)

FXS is a common genetic disorder associated with intellectual disability and autism spectrum disorder (ASD). Patients with FXS exhibit symptoms such as hyperactivity, impulsivity, anxiety, impaired language development, and seizures (Hagerman et al. 2017). FXS is caused by mutations in the *FMR1* gene, which encodes the RNA‐binding protein FMRP that regulates the translation of mRNAs encoding proteins involved in synaptic plasticity and neurodevelopment (Lozano et al. 2014). Expression of the FMR1 gene is suppressed when the CGG trinucleotide repeat in its 5′ untranslated region expands beyond 200 repeats, a threshold that distinguishes the full mutation from normal and premutation alleles (Willemsen et al. 2011). During transcription of the expanded *FMR1* allele, the nascent RNA forms stable RNA–DNA hybrids with the template DNA strand due to the high stability of CGG repeats, thereby suppressing transcription at the *FMR1* locus (Usdin and Kumari 2015).

In addition to transcriptional repression, loss of FMRP impairs neuronal function. Under normal conditions, FMRP represses the translation of specific mRNAs at dendritic spines, thereby regulating synaptic development and plasticity (Darnell et al. 2011; Kao et al. 2010; Zalfa et al. 2003). In the absence of FMRP, this translational repression is relieved, resulting in excessive local protein synthesis at synapses. Such dysregulated translation disrupts synaptic signalling and contributes to the abnormal dendritic spine morphology and cognitive deficits observed in FXS (Darnell et al. 2011). Furthermore, unresolved R‐loops at the FMR1 locus can activate the DDR and induce DNA strand breaks, indicating that persistent R‐loops contribute to genomic instability in FXS (Loomis et al. 2014).

### 5.6 Alzheimer's Disease (ad)

ad is the most common neurodegenerative disorder and is characterized by progressive cognitive decline, synaptic dysfunction, and selective neuronal loss in the hippocampus and cerebral cortex (Chung and Cummings 2000; Friedland et al. 1988). In addition to the hallmark amyloid‐β plaque and tau neurofibrillary tangles, genomic instability and DNA damage accumulation are increasingly recognize as early and pervasive features of ad pathology (Dickson and Vickers 2001; Murray et al. 2011). Neurons affected in ad exhibit exceptionally high transcriptional activity, long gene architectures, and elevated oxidative stress (Foret et al. 2024; Soheili‐Nezhad et al. 2021; Williams et al. 2021). It is hypothesized that oxidative DNA lesions can stabilize RNA–DNA hybrids and impair the recruitment or activity of R‐loop‐resolving factors, leading to persistent R‐loop and transcriptional‐associated DNA damage. Increased RNA–DNA hybrid signals and activation of the DDR have been observed in ad models (Scopa et al. 2023; Zhang et al. 2023). R‐loop accumulation in ad may further exacerbate neuronal dysfunction by interfering with the transcription of long genes involved in synaptic plasticity, calcium signalling, and neuronal survival (Jauregui‐Lozano et al. 2022; Watts et al. 2022). These transcriptional defects may synergize with tau‐mediated chromatin relaxation and impaired DNA repair pathway, thereby amplifying genome instability over time (Asada‐Utsugi et al. 2022; Frost et al. 2014). Although direct causal evidence linking R‐loop to ad initiation remains limited, current data support a model in which R‐loop dysregulation acts as a critical amplifier to transcriptional stress and DNA damage in vulnerable neuronal populations.

### 5.7 Parkinson's Disease (PD)

PD is characterized by the progressive degeneration of dopaminergic neurons in the substantia nigra, resulting in reduced striatal dopamine levels and a motor symptoms, including bradykinesia, rigidity, and resting tremor (Kang et al. 2005; Miller and Cronin‐Golomb 2010; Recasens et al. 2014; Wakabayashi et al. 2007). Dopaminergic neurons are particularly susceptible to oxidative stress due to dopamine metabolism, mitochondrial dysfunction, and high bioenergetic demand conditions that strongly predispose these cells to transcriptional stress and genome instability (Burbulla et al. 2017; Ni and Ernst 2022; Shi et al. 2017).

Oxidative stress has been shown to promote R‐loop stabilization by inducing DNA lesions that favor RNA–DNA hybrid formation and impair their resolution. In PD models, defects in mitochondrial function and redox homeostasis are therefore hypothesized to indirectly enhance R‐loop accumulation at actively transcribed neuronal genes (Imaizumi et al. 2012; Pfeifer 2024; Silva et al. 2018). In parallel, mutations in PD‐associated genes such as LRRK2, PARK7 (DJ‐1), and PINK1 have been linked to impaired DNA repair capacity and altered transcriptional regulation (Blauwendraat et al. 2020; Chen et al. 2020; Imberechts et al. 2022; Vázquez‐Vélez and Zoghbi 2021; Wang et al. 2023), potentially exacerbating R‐loop associated DNA damage. Recent studies have reported elevated DNA damaged markers and transcription‐coupled repair defects in dopaminergic neurons in PD, supporting a role for genomic instability in disease progression (Anwer et al. 2025; Khan et al. 2025; Milanese et al. 2018; Sepe et al. 2016). Although direct detection of pathological R‐loops in PD patient tissue remains limited, the combination of oxidative stress, impaired DNA repair, and long gene transcription suggests that R‐loop accumulation may represent an underappreciated contributor to dopaminergic neuron vulnerability.

### 5.8 Aicardi–Goutières Syndrome (AGS)

AGS is a type I interferonopathy that causes a spectrum of neurologic impairment affecting both the brain and the immune system (Gavazzi et al. 2025). It is a rare autosomal recessive neurodevelopmental disease that mimics congenital viral infection and is characterized by progressive microcephaly, intracranial calcifications, leukodystrophy, seizures, spasticity, and severe intellectual disability (Aicardi and Goutieres 1984; Crow et al. 2006). AGS is caused by mutations in genes involved in nucleic acid metabolism and sensing, most notably *RNase H2* (Al Mutairi et al. 2018; Piccoli et al. 2021). Regarding R‐loop dynamics, mutations in *RNase H2* impair the resolution of RNA–DNA hybrids, leading to prolonged R‐loops that activate innate immune pathways and chronic interferon (Pizzi et al. 2015; Secchi et al. 2024). In AGS, unresolved R‐loops drive chronic activation of DDRs and innate immune signalling, ultimately resulting in persistent type I interferon production (Giordano et al. 2022). This chronic interferon signalling contributes to neuroinflammation and progressive neurological dysfunction.

### 5.9 Coffin–Siris Syndrome (CSS)

CSS is a rare congenital disorder characterized by developmental delay, intellectual disability, coarse facial features, hypoplasia or absence of the fifth fingers or toenails, and variable brain malformations (Levy and Baraitser 1991; Yu et al. 2022). CSS is caused by heterozygous mutations in genes encoding components of the SWI/SNF (BAF) chromatin‐remodelling complex, including ARID1B, SMARCA4, SMARCB1, and SMARCE1 (Tsurusaki et al. 2012). During brain development, the SWI/SNF complex plays a critical role in nucleosome remodelling and transcriptional regulation. Mutations in these genes impair chromatin accessibility and disrupt transcriptional control of target genes involved in neurogenesis, cell cycle regulation, and DNA repair (Chmykhalo et al. 2025). Emerging evidence also suggests that SWI/SNF dysfunction may promote aberrant R‐loop formation (Bayona‐Feliu et al. 2021; Davó‐Martínez et al. 2023). The chromatin‐remodelling activity of the SWI/SNF complex affects both DNA topology and transcription elongation, processes that are closely linked to R‐loop homeostasis. Experimental studies have shown that loss of specific BAF components leads to the accumulation of R‐loops at defined genomic loci, which can interfere with transcriptional progression (Tsai et al. 2021). In CSS, impaired chromatin remodelling may therefore increase R‐loop‐associated genomic instability, particularly during early neurodevelopment. This genomic instability likely contributes to the neurodevelopmental defects and structural brain abnormalities observed in affected individuals. Taken together, these findings suggest that dysregulated R‐loop metabolism may represent a key mechanistic link between SWI/SNF dysfunction and the pathogenesis of CSS.

### 5.10 Huntington's Disease (HD)

HD is an autosomal dominant neurodegenerative disorder characterized by progressive motor dysfunction, cognitive decline, and psychiatric disturbances (Louis et al. 1999; Nance and Sanders 1996; Wilson and Garron 1980). The disease is caused by an expansion of a CAG trinucleotide repeat in exon 1 of the *huntingtin* (*HTT*) gene (Mangiarini et al. 1996; Persichetti et al. 1995; Warby et al. 2009). Although mutant HTT protein toxicity has long been considered the primary pathogenic mechanism (Gao et al. 2019), increasing evidence indicated that transcription‐associated genome instability contributes significantly to neuronal vulnerability in HD (Goula et al. 2012). The expanded CAG repeat is intrinsically prone to R‐loop formation during transcription, due to its repetitive sequence composition and impaired transcriptional elongation (Kaalak et al. 2014). R‐loop formed at these expanded tracts stabilize RNA–DNA hybrids and expose ssDNA, creating substrates for DNA damage and aberrant repair. Experimental studies have demonstrated that transcription across expanded CAG repeats promotes R‐loop accumulation, which in turn induces DSB and activates the DDR (Lin and Wilson 2012; Sledzinski et al. 2024; Su and Freudenreich 2017). These effects are particularly detrimental in post‐mitotic neurons, where reliance on transcription‐coupled DNA repair is high and repair capacity is limited. In addition to locus‐specific effects, HD neurons exhibit widespread transcriptional dysregulation and chromatin alterations that may further enhance R‐loop formation genome‐wide. Mutant *HTT* has been shown to interfere with transcriptional regulators and DNA repair factors, potentially impairing R‐loop resolution and amplifying transcription‐associated DNA damage (Dunah et al. 2002; Enokido et al. 2010; Gao et al. 2019). Consistent with this model, increased DNA damage markers and defective repair responses have been observed in HD patient brains and cellular models (Askeland et al. 2018; Jeon et al. 2012; Palminha et al. 2022). Overall, these findings support a model in which R‐loop dysregulation acts as a critical mediator linking CAG repeat expansion, transcriptional stress, and genome instability to selective neuronal degeneration in HD.

## 6 Advancements in R‐Loop Detection and Therapeutic Approaches

The development of methods to artificially modulate R‐loop formation and resolution represents a promising strategy for treating the neurological diseases associated with transcriptional stress and genome instability. Identification of small‐molecule compounds that stabilize or resolve R‐loops could potentially alleviate transcriptional stress and DNA damage (Marinello et al. 2013). For example, enhancing the enzymatic activity of topoisomerase 1 or RNase H may selectively reduce excessive R‐loop accumulation, thereby protecting neurons from DNA damage.

Given the direct involvement of RNase H2 in AGS pathogenesis, strategies that compensate for impaired RNA–DNA hybrid resolution represent a rational therapeutic approach. Overexpression of RNase H1 has been shown to suppress R‐loop accumulation and attenuate DDRs in cellular models (Cristini et al. 2022), suggesting that functional redundancy among RNase H enzymes could be therapeutically exploited in RNase H2–deficient contexts. In addition, upregulation or stabilization of R‐loop–resolving helicases, such as SETX, may further alleviate transcription‐associated genomic stress (Rao et al. 2024). Although these approaches broadly reduce R‐loop burden, careful consideration is required to avoid disrupting physiological R‐loops that are essential for normal transcriptional regulation.

More refined therapeutic strategies aim to selectively eliminate pathological R‐loops while preserving normal R‐loop function. A recently developed approach employs catalytically inactive Cas9 (dCas9) fused to RNase H1, enabling targeted degradation of RNA–DNA hybrids at defined genomic loci without inducing DSBs (Abraham et al. 2020). In the context of AGS, such locus‐specific targeting could be particularly advantageous for suppressing R‐loops that trigger cytosolic nucleic acid sensing or activate aberrant DDRs, thereby limiting downstream interferon signalling (Giordano et al. 2022; Park et al. 2021). Therapeutic approaches targeting downstream interferon pathways, including JAK–STAT inhibition, may therefore complement R‐loop–directed strategies (Frémond et al. 2023; Vanderver et al. 2020). By reducing interferon‐mediated neuroinflammation, such interventions could mitigate secondary neuronal damage, particularly in post‐mitotic neurons, where DNA repair capacity is intrinsically limited (Barrett et al. 2020).

Additionally, therapeutic approaches that modulate transcription or RNA processing, including antisense oligonucleotides (ASOs), have shown promise in correcting disease‐associated gene expression defects, although their effects are not necessarily mediated through direct regulation of R‐loop (Wang et al. 2022).

Recent advances in R‐loop detection technologies have rapidly expanded our understanding of their roles in neurons. Techniques such as DRIP‐seq (DNA–RNA immunoprecipitation sequencing) (Ginno et al. 2013), R‐loop CUT&RUN (targeted DNA cleavage using nucleases) (Yan et al. 2019), R‐loop CUT&Tag (targeted cleavage and tagging) (Wang et al. 2021), RIAN‐seq (R‐loop identification assisted by nucleases and sequencing) (Li et al. 2025), R‐ChIP (reverse chromatin immunoprecipitation) (Chen et al. 2019) and MapR (Yan and Sarma 2020) have improved the accuracy of genome‐wide R‐loop mapping. These approaches have enabled the identification of R‐loop‐prone sites within the neuronal genome and facilitated the correlation of their presence with disease‐relevant genomic regions, thereby advancing our understanding of disease mechanisms. Mapping R‐loop profiles in patient‐derived neurons is expected to further clarify how R‐loop dysregulation contributes to neurological damage.

Numerous studies have reported correlations between altered R‐loop dynamics and disease progression in neurodegenerative disorders such as Alzheimer's and PD. Continued research focused on developing methods to modulate R‐loops holds considerable therapeutic potential. Moreover, studies using animal models will deepen our understanding of R‐loop functions in vivo, potentially revealing broader biological roles and facilitating the development of novel treatments for additional neurological diseases.

## 7 Epilogue

Why is R‐loop dysregulation particularly relevant to neurological diseases? Several intrinsic features of neurons render them especially susceptible to R‐loop dysregulation. We propose this vulnerability arises from the convergence of three key factors; post‐mitotic status, sustained transcriptional demand, and long cellular lifespan. First, because neurons are post‐mitotic and do not proliferate, DNA damage, such as DSBs, caused by the accumulation of R‐loops cannot be resolved through cell division (Shelly et al. 2018; Welty et al. 2018). Consequently, DNA lesions accumulate over time, contributing to chronic neurodegeneration. This limitation heightens the dependence on accurate DNA repair pathways, which are often compromised in neurological disorders.

Second, neurons are long‐lived and functionally complex, requiring precise and sustained gene expression (Bhat et al. 2022; Eade et al. 2012). Their exceptionally high transcriptional activity, particularly of long and neuron‐specific genes, increases the likelihood of R‐loop formation, which can disrupt transcriptional fidelity, RNA processing, and epigenetic regulation (Jauregui‐Lozano et al. 2022; Zhang et al. 2024). Although physiological R‐loops play critical roles in gene regulation, RNA splicing, and transcription termination (as noted in the introduction), dysregulated R‐loops can misexpress neuron‐specific genes and impair RNA maturation. Furthermore, R‐loop‐induced DNA damage can activate innate immune responses, promoting chronic neuroinflammation—a hallmark of several neurological disorders with inflammatory components (Crossley et al. 2023). Prolonged activation of DNA damage and inflammatory signalling may exacerbate neuronal dysfunction, creating a feed‐forward loop of genomic stress. Importantly, recent studies have shown that many genes associated with neurological diseases are directly involved in R‐loop formation and resolution. Disease‐specific mutations may selectively disrupt R‐loop regulation at particular genomic loci or transcriptional programs characteristic of specific neuronal subtypes (Figure 2).

For example, mutations in genes encoding RNA‐binding proteins (e.g., FMRP in FXS), nucleases (e.g., RNase H2 in AGS), and chromatin remodels (e.g., components of BAF complex in CSS) perturb R‐loop homeostasis through distinct yet interconnected mechanism. These alterations affect neuronal function via multiple pathways, including transcriptional interference, genomic instability, epigenetic dysregulation, and neuroinflammation. Thus, neurological disorders associated with R‐loop regulators reflect the selective vulnerability of neuronal gene networks that rely on tightly controlled R‐loop dynamics. Elucidating how physiological R‐loop transition into pathological structures is critical for understanding disease mechanisms and developing strategies to restore R‐loop homeostasis.

## Author Contributions

**Nur Rasyiqin Rasli:** conceptualization, investigation, visualization, writing – original draft, writing – review and editing. **Yu Katsuyama:** conceptualization, writing – review and editing, supervision.

## Funding

The authors have nothing to report.

## Conflicts of Interest

The authors declare no conflicts of interest.

## Data Availability

No data were used for the research described in the article.
